# Supplementary material for: Ecological drivers of ultraviolet colour evolution in snakes
Source: Nat Commun. 2024 Jun 18;15:5213. doi: 10.1038/s41467-024-49506-4 (PMC11189474; doi:10.1038/s41467-024-49506-4)
Supplement: Supplementary file 1 — Supplementary Information [file 41467_2024_49506_MOESM1_ESM.pdf]

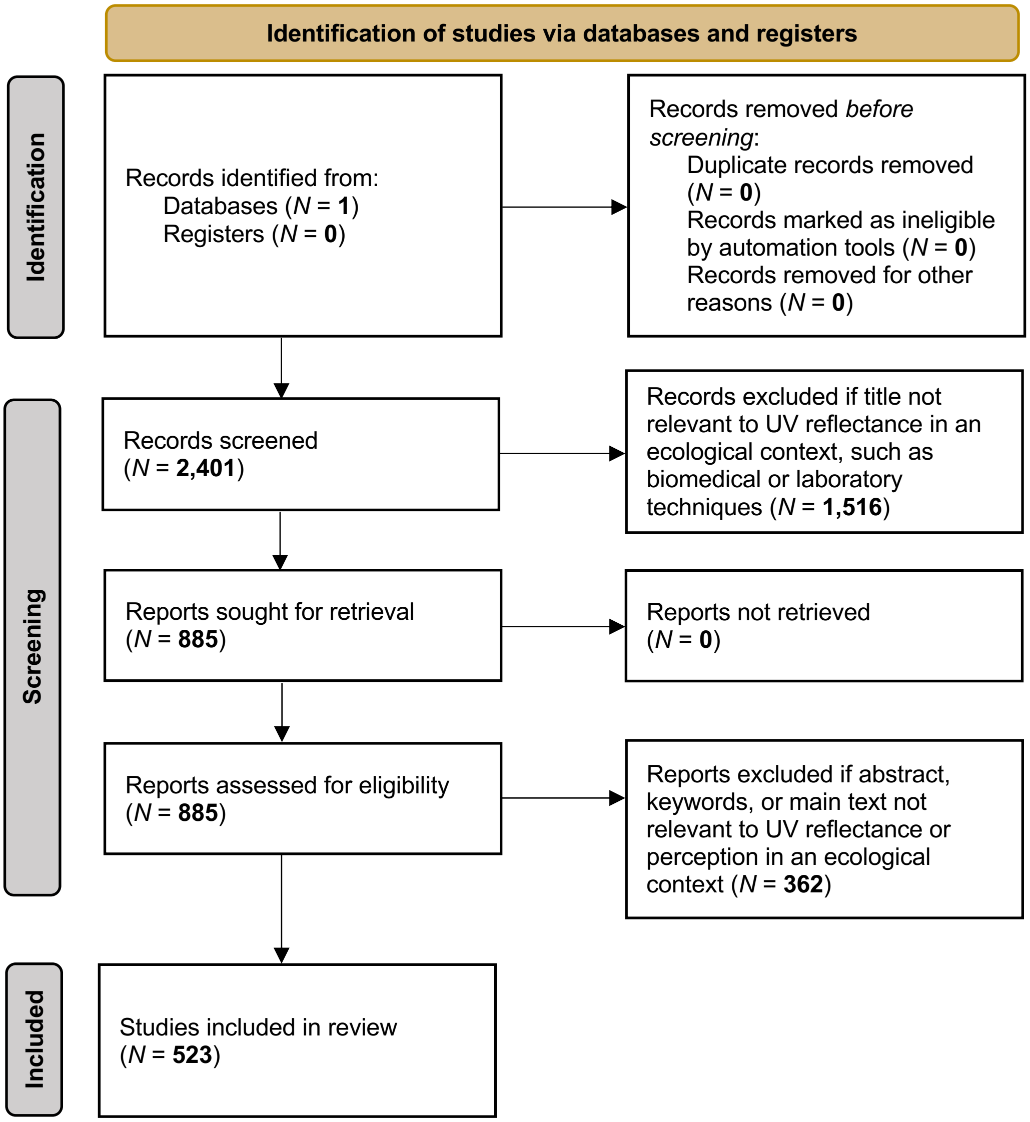

Supplementary Fig. 1. PRISMA flowchart depicting the literature search and filtering steps used for our review of relevant published research on UV colouration in living organisms. We searched the *Scopus* online citation database using the search string *ultraviolet OR uv OR "nectar guide" AND (reflect* OR absorb* OR color* OR colour* OR pattern* OR signal*) AND ("natural selection" OR "sexual selection" OR adapt*)* and limited the results to articles and reviews in the subject areas of 1) biochemistry, genetics, and molecular biology, 2) agricultural and biological sciences, and 3) environmental science. We then filtered the results for those that pertained to UV reflectance, colouration, or perception in an ecological context, 511 of which mapped to taxonomic categories in Fig. 1 of the main manuscript.

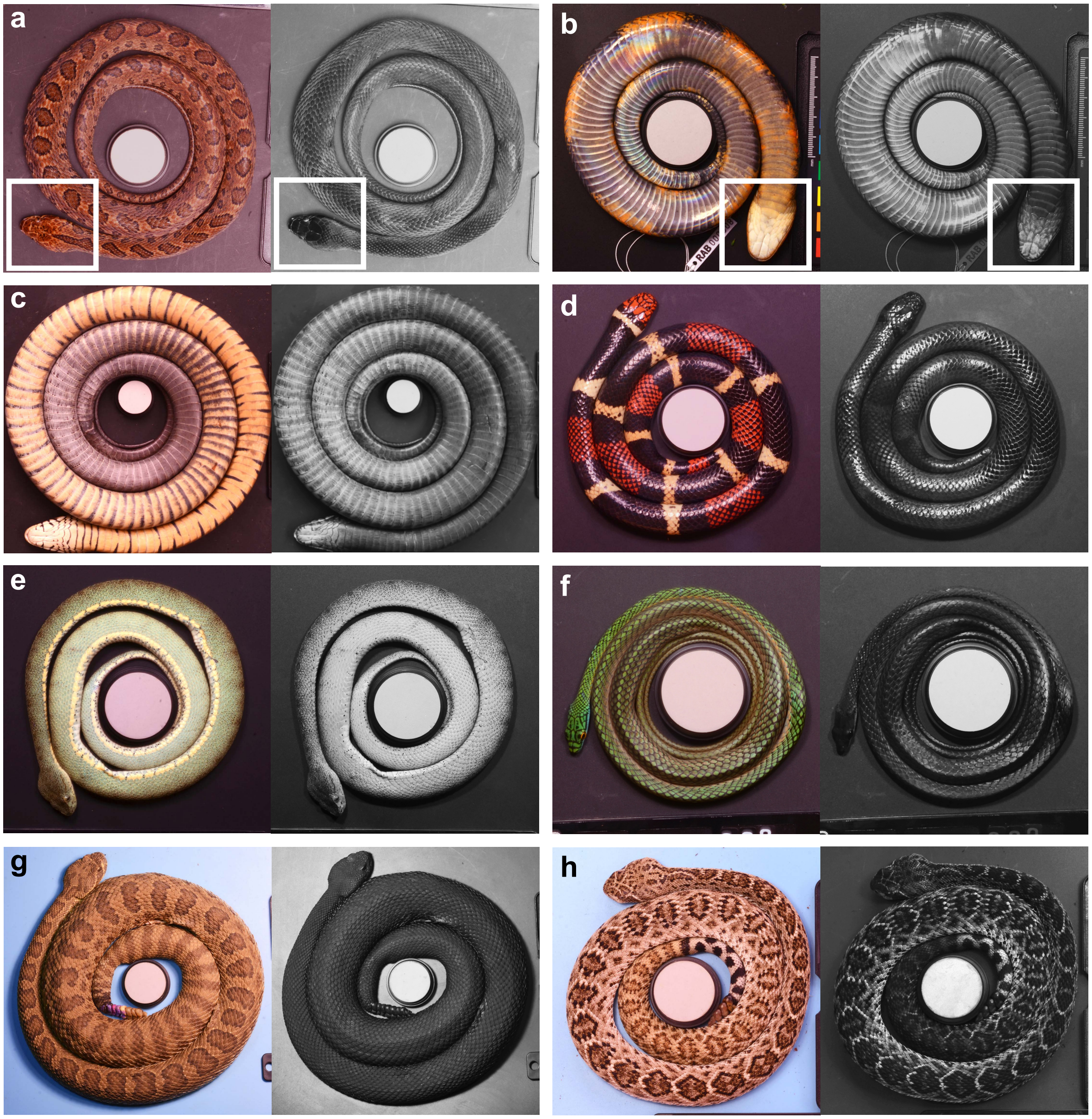

Supplementary Fig. 2. Widespread variation in UV reflectance across snakes. White boxes highlight areas of interest in each photo, such as the appearance or disappearance of patterns between visible (left) and UV (right) photos for each specimen. **a**, Dorsal view of *Leptodeira rhombifera* (RAB-3241/UMMZ-247098). **b**, Ventral view of *Xenodon severus* (RAB-0608/UMMZ-248367). **c**, Ventral view of *Drymarchon melanurus* (RAB-3059/UMMZ-247140). **d**, Dorsal view of *Micrurus surinamensis* (RAB-1099/UMMZ-246861). **e**, Dorsal view of *Bothrops bilineatus* (RAB-1382/UMMZ-246865). **f**, Dorsal view of *Leptophis ahaetulla* (RAB-0933/UMMZ-246823). **g**, Dorsal view of *Crotalus viridis* (HLC-7420, not vouchered). **h**, Dorsal view of *Crotalus atrox* (RAB-3657/UMMZ-249110).

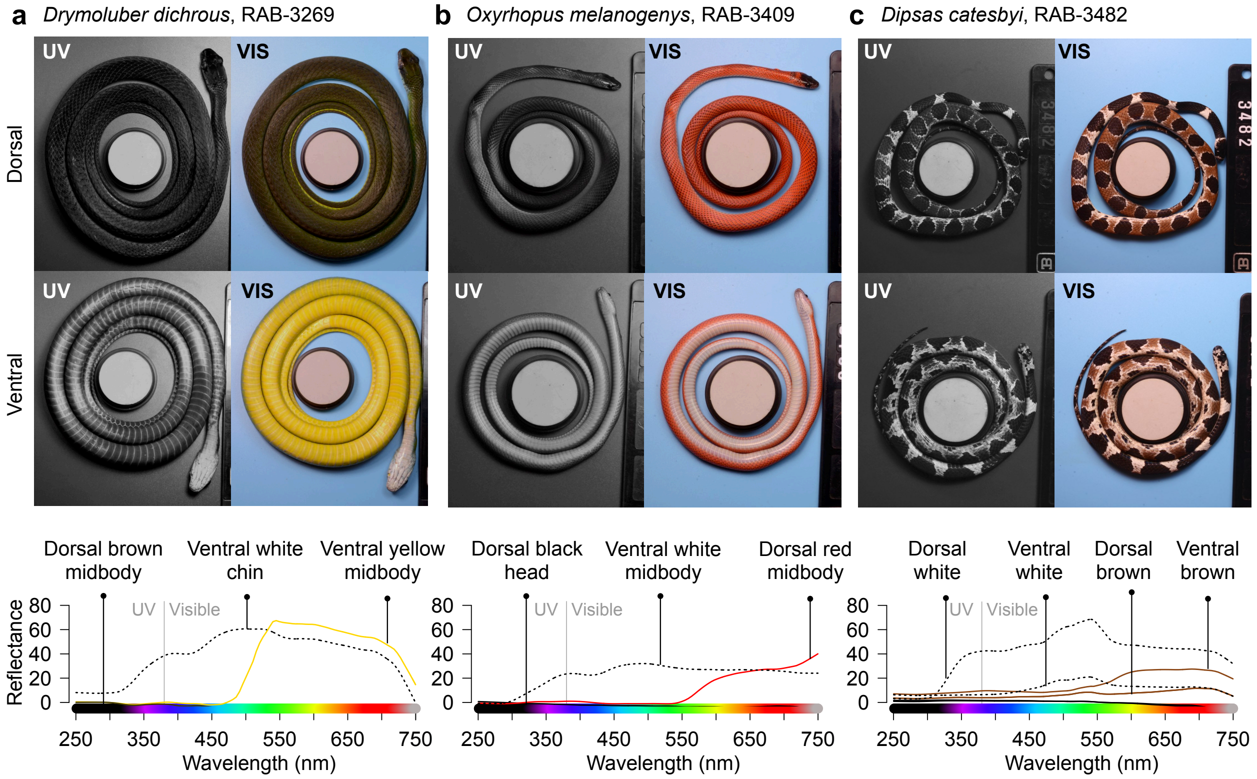

Supplementary Fig. 3. Colour patch photographic data match spectrometer measurements. a-c, Patch measurements of the dorsal (upper) and ventral (middle) colours from multispectral photographs correspond to spectrometer measurements of reflectance (below). Note significant specular reflectance (not UV reflectance) on the shiny ventral scales of **a** - see also Supplementary Fig. 6 for further treatment of specular reflectance on shiny surfaces. Source data are provided as a Source Data file.

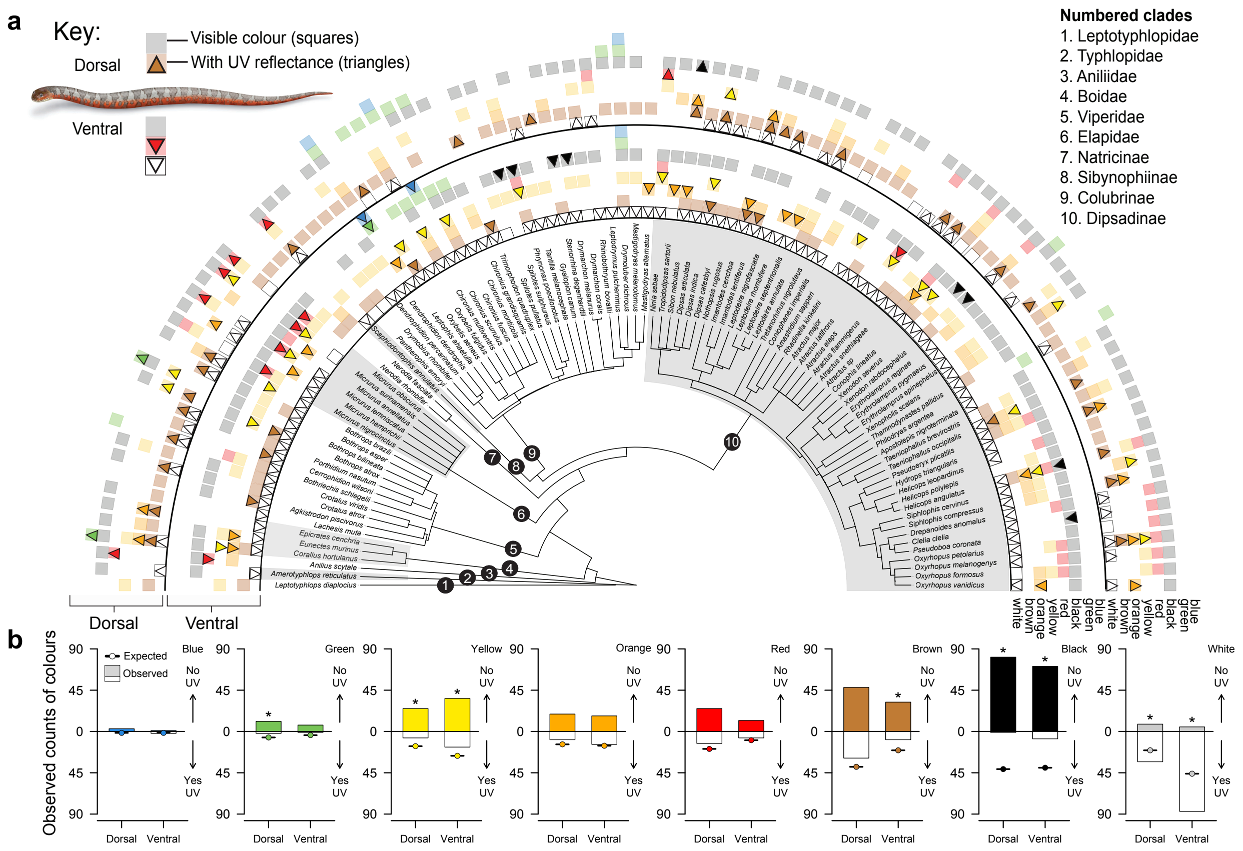

Supplementary Fig. 4. Phylogenetic distribution of UV reflectance by species, body surface, and visible colour. a, Note that this tree is identical to Fig. 2, but displays species name tip labels. Visible colours are displayed with shaded squares, and the lack of a square for a colour/ species combination means that we measured no individuals with that visible colour on that body surface (dorsal vs. ventral). Additional UV reflectance in a patch of a colour is indicated by an embedded triangle, pointing upwards for dorsal surface and downwards for ventral surfaces. For reference, the snake in the key (*Helicops angulatus*) has two dorsal visible colours (grey and brown) and only one of them has UV reflectance (brown). This snake has three ventral visible colours (grey, red, and white) and two of them have UV reflectance (red, white). However, this species is highly variable, and its full visible and UV colour profile is shown by its tip state within clade 10. Scientific illustration of *Helicops angulutus* in the graphical legend courtesy of J. Megahan. **b**, Observed counts of UV reflectance (bottom bar plots) for each colour are generally lower than expected values (points overlaying lines) under a random distribution (observations of each colour are plotted by whether or not they reflect UV light, bottom vs. top panels). Asterisks indicate a significant deviation from the null expectation under a Chi-squared test at $P < 0.025$, which is adjusted via Bonferroni correction to account for non-independence of dorsal and ventral data. However, this pattern is reversed for white colouration, in which UV reflectance is far more common than expected. Note that every colour has at least one observation of UV reflectance, and that its distribution across the visible spectrum suggests that nothing inherently constrains UV reflectance to only colours with peaks nearer to 360nm. Source data are provided as a Source Data file.

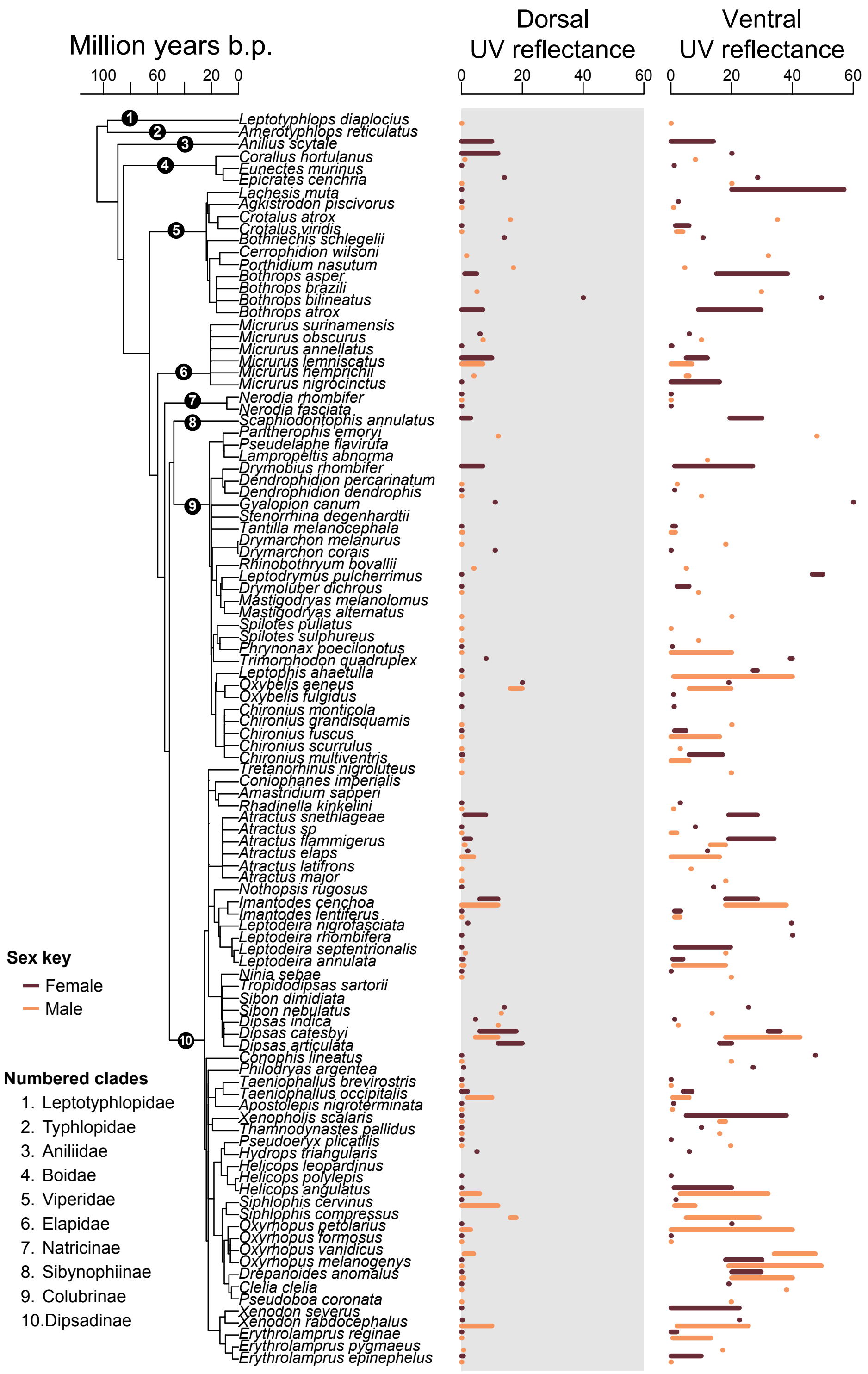

Supplementary Fig. 5. Variation in UV reflectance within species does not vary by sex. Note that this tree is identical to Fig. 3 in the main manuscript, but displays species name tip labels. Source data are provided as Supplementary Data 2.

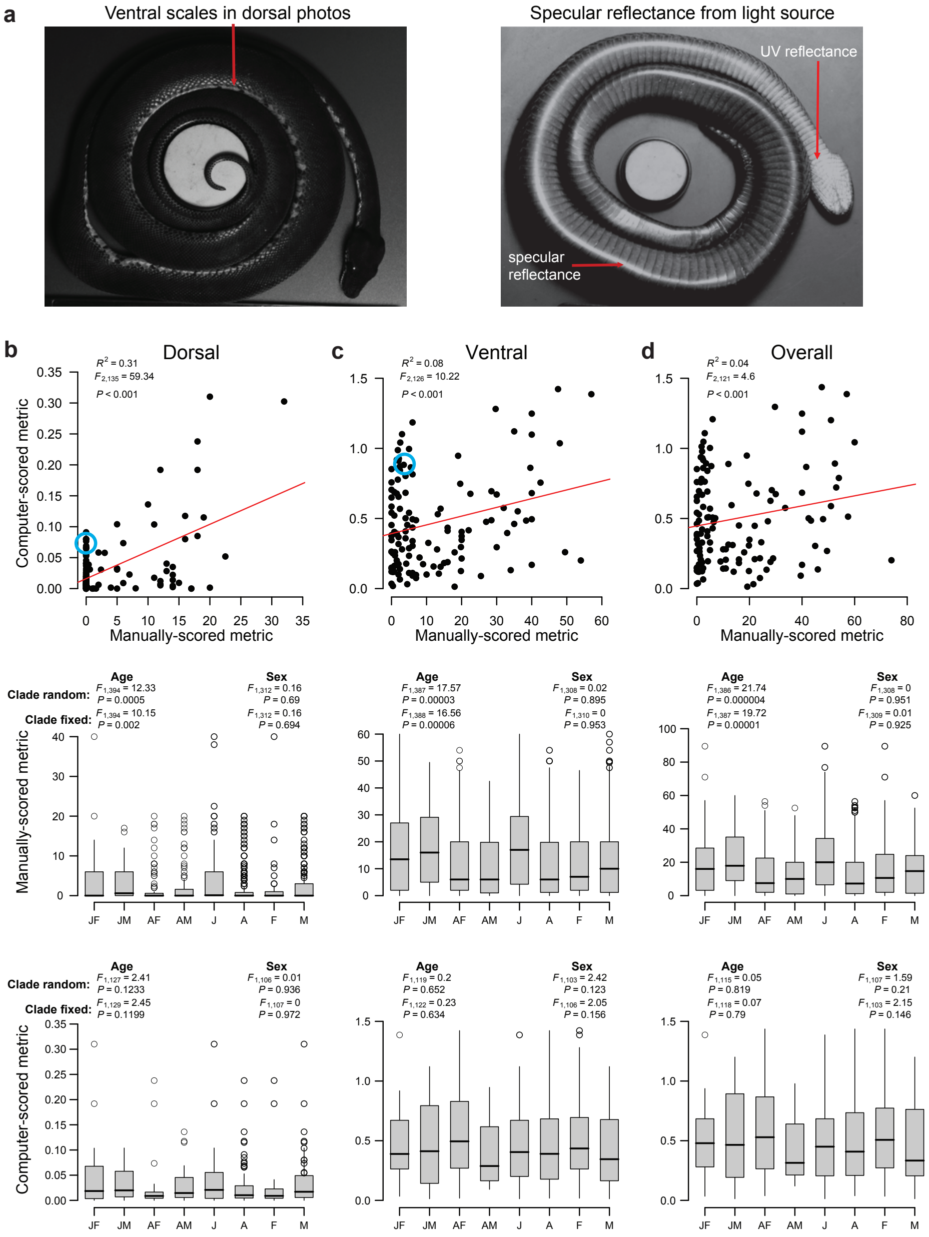

Supplementary Fig. 6. Two different approaches to measuring UV reflectance on all individuals yield similar results. a, Positioning and specular reflectance from shiny scales can create discordance in UV scoring between humans and computers, although the two metrics are positively correlated overall (**b-d;** compare middle to bottom row). Left image is a dorsal photo with visible ventral scales with high reflectance. Human observers easily excluded ventral scales for dorsal scoring, but they were counted by the computer as falsely-positive for dorsal UV reflectance (blue circle in **b**). Right image is a ventral photo with high specular reflectance erroneously scored as UV reflectance by the computer (blue circle in **c**). Box plots (centre line is data median, bounds represent ± 1.5 IQR, and whiskers represent minima and maxima excluding outliers) show dorsal, ventral, and overall UV reflectiveness values for juveniles (J), adults (A), females (F), and males (M) for both manually-scored (middle row) and computer-scored metrics (bottom row). Source data are provided as a Source Data file and Supplementary Data 2.

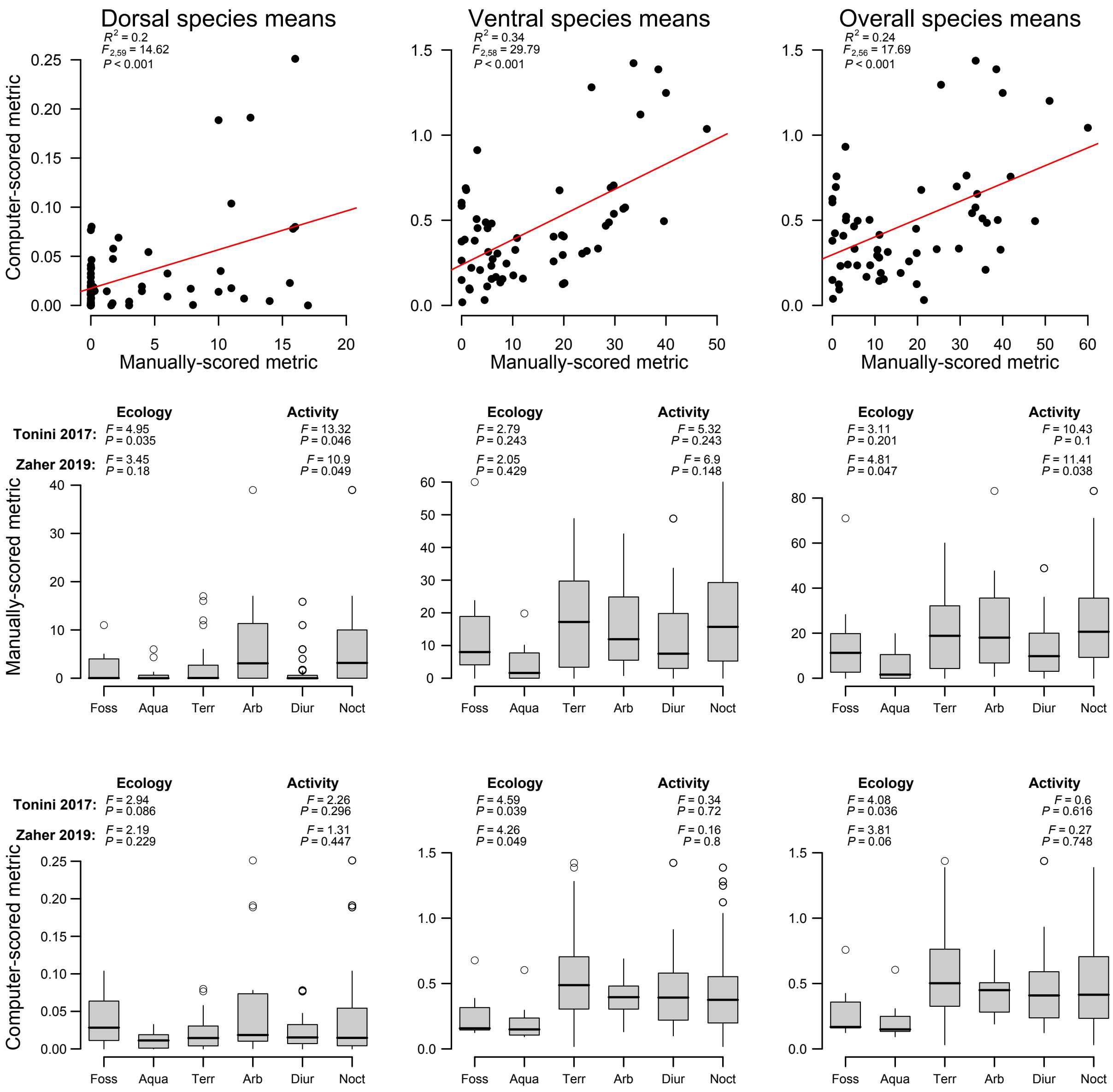

Supplementary Fig. 7. Species mean comparison of manually-scored and computer-scored UV reflectance metrics yield similar results. For species means (as opposed to individual snake measurements in Supplementary Fig. 6), both metric box plots (centre line is data median, bounds represent ± 1.5 IQR, and whiskers represent minima and maxima excluding outliers) generally capture similar patterns for all comparisons (compare middle row to bottom row). Source data are provided as a Source Data file.

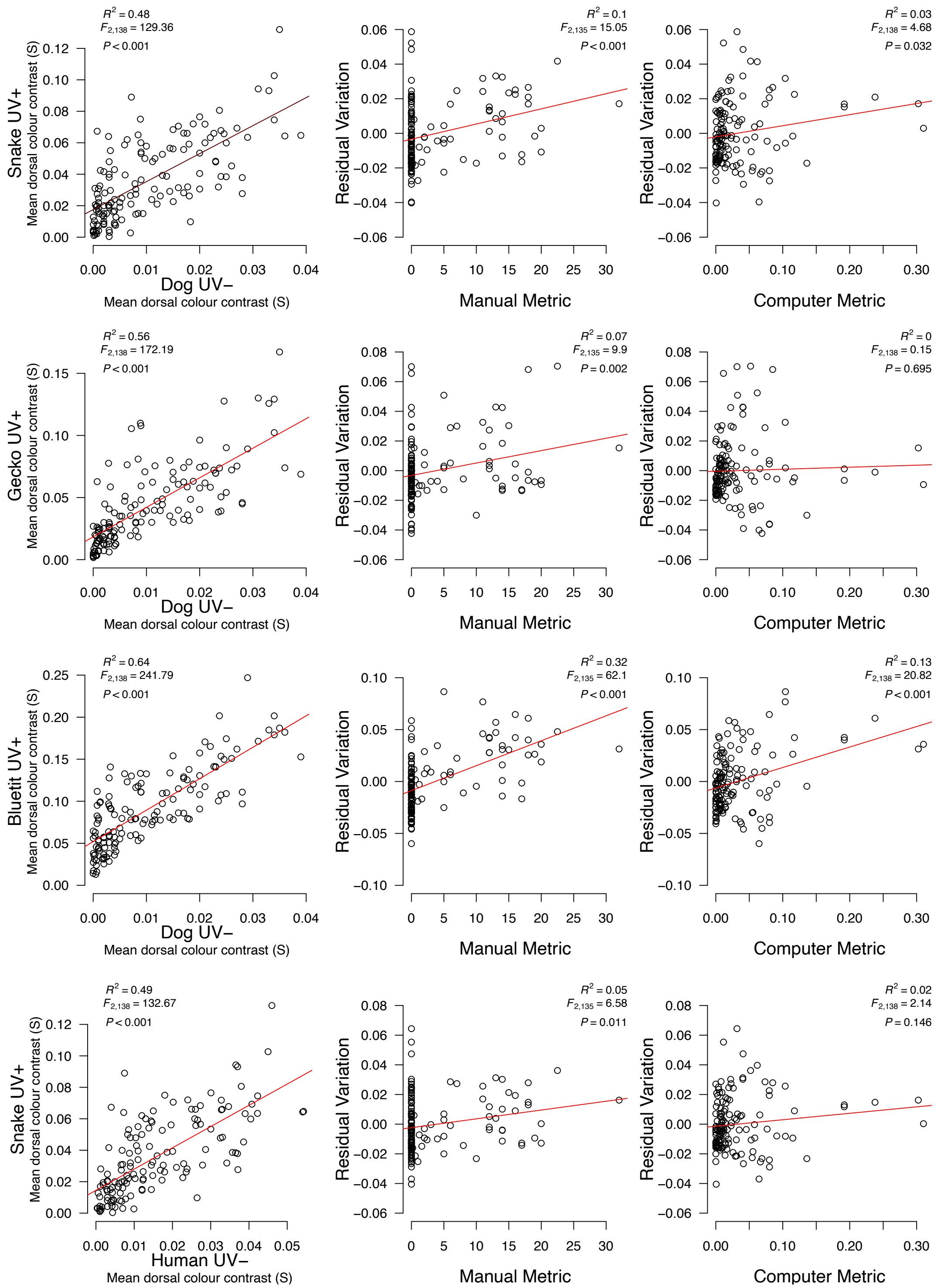

Supplementary Fig. 8. Receiver comparisons (by row) show proportion of variance explained by UV reflectance. Left: Regressing mean colour contrast values (ΔS, a measure of conspicuousness) of the same snake between pairwise UV-weak to UV+ receivers generates residual variation that can be regressed against two independent methods for quantifying UV reflectance (middle and right). Continued in Supplementary Fig. 9; see also Fig. 4c, d in the main manuscript. Source data are provided as a Source Data file.

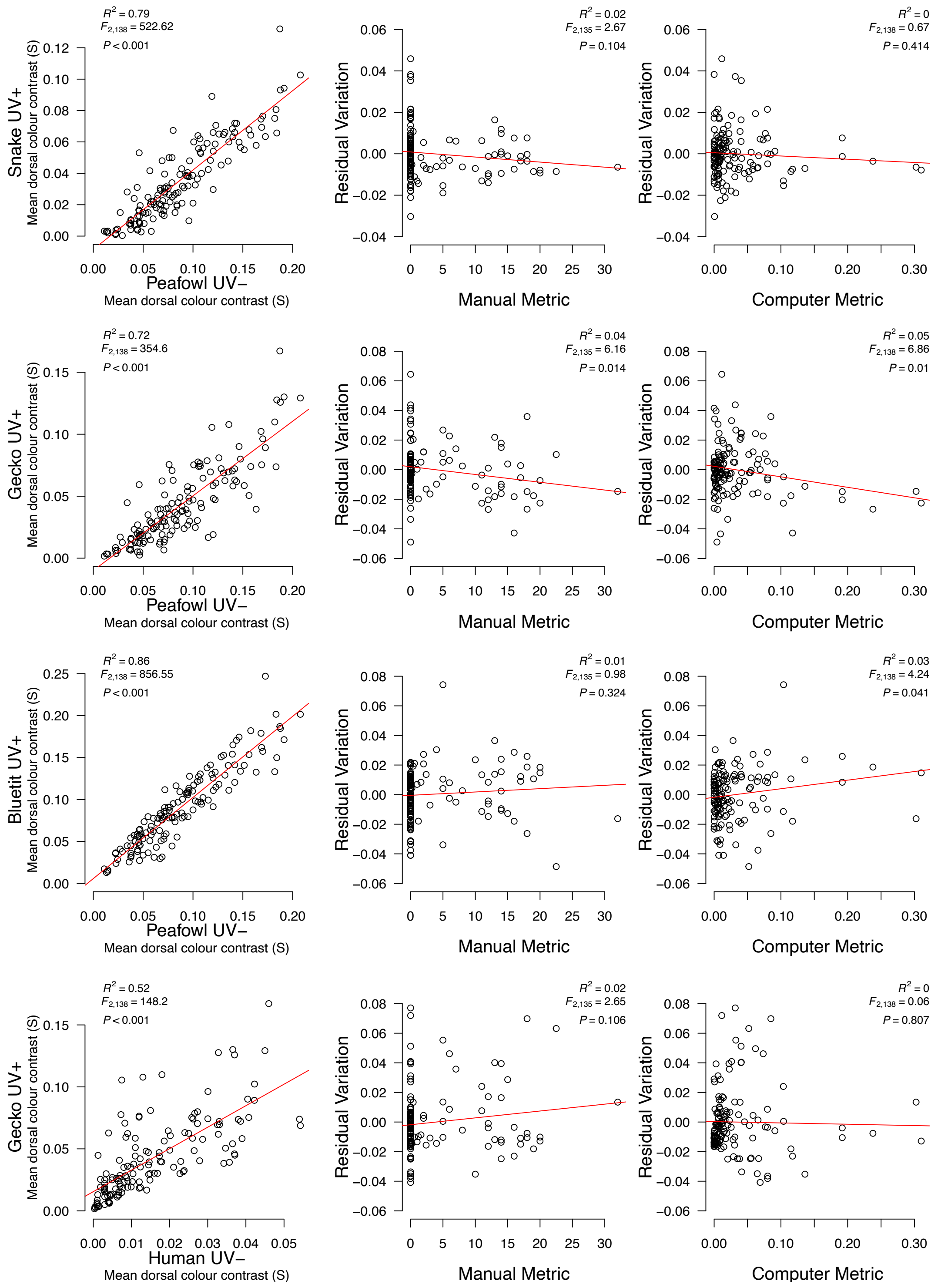

Supplementary Fig. 9. Receiver comparisons (by row) show proportion of variance explained by UV reflectance. Left: Regressing mean colour contrast values (ΔS, a measure of conspicuousness) of the same snake between pairwise UV-weak to UV+ receivers generates residual variation that can be regressed against two independent methods for quantifying UV reflectance (middle and right). Continuation from Supplementary Fig. 8; see also Fig. 4c, d in the main manuscript. Source data are provided as a Source Data file.

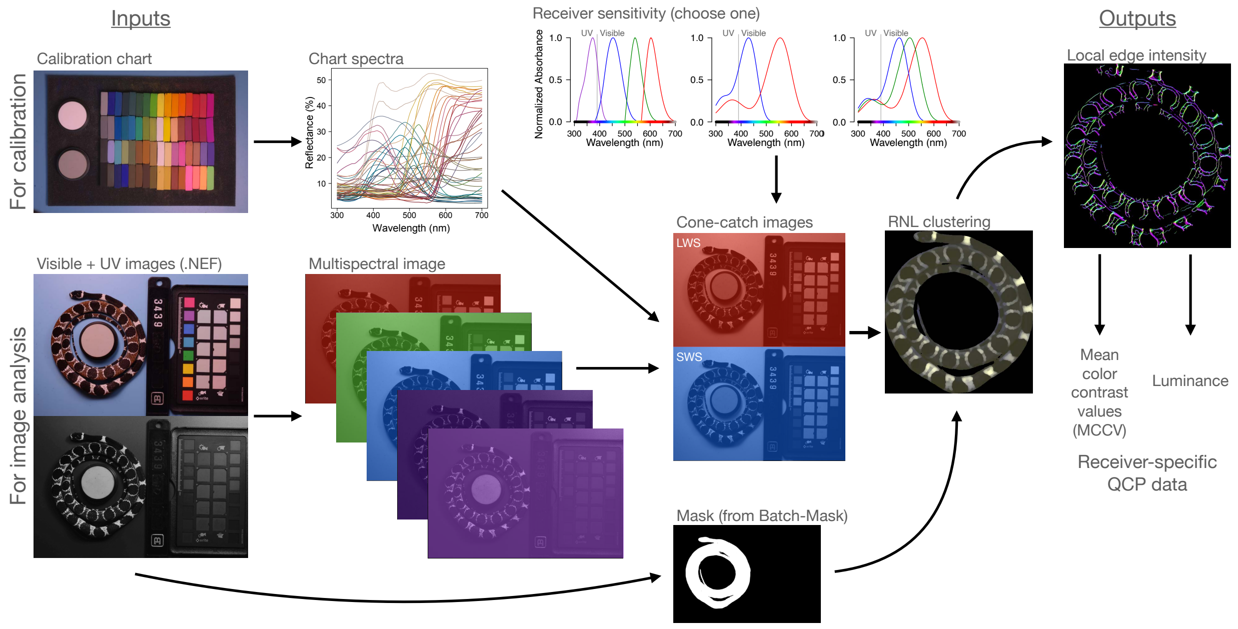

Supplementary Fig. 10. Work flow demonstrating how colour pattern metrics for non-human visual systems were calculated from UV and human-visible image pairs. First, we calibrated our camera by imaging a chart of commercially available artists' pastels and measuring their reflectance using the 'Measure chart' function in micaToolbox. These values were combined with spectral reflectances of each pastel and known spectral sensitivity data for different viewers to produce cone-catch models using the 'Generate cone catch model from chart' function (one cone-catch model per viewer/camera combination). Images were combined into multispectral (5-channel) images and normalized to 40% grey standard in each image using the 'Generate multispectral image' function, and converted to cone-catch images using the 'Convert to Cone Catch' function. Images were scaled using the 50mm scalebar in each image, and a mask for the snake in the image as generated by Batch-Mask was imported as a region of interest. We then ran the 'Quantitative Color Pattern Analysis' (QCPA) pipeline to cluster the image based on colours distinguishable to that viewer (receptor noise limited clustering). Finally, we ran the 'Local Edge Intensity Analysis' tool of the QCPA pipeline to generate edge intensity values for colour and luminance on the snake. Source data for chart spectra provided as Source Data file.

Supplementary Note 1: Potential effects of skin and scale properties on UV reflectance

The snakes that we collected from natural populations were single point encounters of individuals with unknown histories, which necessarily prevented us from directly controlling for some factors that might affect patterns of UV reflectance in snakes. Thus, we discuss below four main ways in which variation in physical properties of skin and scales across developmental time points could influence our results. Overall, we viewed these issues as unavoidable sources of potential noise in our data, and we designed our sampling to span multiple years, seasons, and environmental conditions to simply distribute such potential effects stochastically. We also note that these issues exist for the study of every colour produced on snake scales rather than being something specific to UV wavelengths, including classic colours commonly studied in snake ecology (*e.g.*, hue of red in aposematic coral snakes[1, 2]). However, some properties that we discuss below have the potential to vary non-randomly across life stages and ecologies, which could weaken the inference that UV colouration is under selection by predators.

First, the thickness of skin and scales increases with body size in snakes, so the thinner scales of juveniles could allow more UV reflectance and influence our ontogenetic result (main manuscript Fig. 3). However, we found the most widespread UV colouration on the ventral scales of snakes, which are the largest and thickest scales across all habitat ecotypes and body sizes, as they are subjected to the highest levels of friction with substrates and are integral to successful locomotion[3]. Thus, thin scales and skin are unlikely to underlie either the finding that juveniles had higher UV reflectance than adults or the finding that ventral reflectance was higher than dorsal reflectance across all age classes.

Second, shed cycles vary across age classes, with juvenile snakes shedding more frequently than adults to accommodate faster growth. Studies on both lizards and birds have shown that individuals that have recently shed or moulted have higher UV reflectance than those that have not shed for an extended period[4, 5]. The only way to directly test this effect in free-living snakes would be to recapture the same individuals at multiple time points (not feasible in the tropics[6]), but it could and should be done on captive snakes. However, in none of these reported cases does shedding or moulting make an individual transition from appearing fully UV reflective to fully unreflective or vice versa, but rather just modulates the brightness of the reflective patches. We also note that dorsal and ventral scales on the same snake are all at the same point in that individual's shed cycle, and yet both surfaces often showed substantial variation in UV reflection both within (*e.g.*, only on chins, Fig. S2a) and between them (reflective venters on snakes with unreflective dorsums, main manuscript Fig. 3a, left). This result is thus unlikely to be explained by something as simple as an individual's overall shed state. Overall, we do expect that there was some effect of shed cycle variation on our brightness results, but view this impact as insufficient to account for the major axes of variation in presence vs. absence of UV reflectance across the snake radiation.

Third, the cellular mechanisms responsible for producing UV reflectance may be correlated with a visible colour that itself is under selection, which would suggest our results could be a by-product of selection in the visible spectrum. We did measure white colours as UV reflective more often than any other visible colour (Fig. 2c, Fig. S4b; note that what humans perceive as "white" generally represents high reflectance across all visible wavelengths, see Fig.

S2). But, we do not yet have enough data on genetic or cellular mechanisms of colour production in snakes to assign structural or pigment-based mechanisms to most colours other than those produced by melanins (blacks) or pteridines (reds; with minimal to no carotenoids[1]), including UV or white. In lizards, it appears at least some UV reflectance is caused by structural colours (specifically iridophores) rather than by pigments[7], and this mechanism of a structural UV colour overlaying pigments of any variety would be consistent with our finding that many visible colours can have UV reflectance, including black (Fig. S1c; Fig. S4b; Fig 2c). Likewise, it is possible that when UV reflectance is not present, it may be due to masking by other pigment types (as in male grouse[8]). While we cannot currently perform a formal test of this hypothesis, we consider the cellular mechanisms underlying UV colour to be the next big frontier in this field necessary to resolve some of these outstanding questions.

Finally, the specular reflectance that results from "shine" on snakes depends on scale type (keeled scales are much less shiny than smooth scales) and amount of oils in snake skin layers, which both vary by species and habitat ecology. Shine or gloss is a known problem in quantitative colour analyses across many taxa (*e.g.*, beetle carapaces that are so shiny that they cannot be photographed in a way that adequately captures their colour profile[9]). We did our best to both minimise and standardise the production of specular reflectance in our illumination setup (see main manuscript Methods), and the species in our dataset included both keeled and smooth scales in each of the four ecological habitat types, especially arboreal (*e.g.*, the arboreal *Bothrops bilineatus* in Fig. S2e is highly keeled with no shine at all, but still highly UV reflective). This standardisation of directionality allowed human observers to simply avoid those patches as having "no scoreable data", while the computer scores did include specular reflectance as UV reflectance. One test of whether confounding effects of specular reflectance drove our ecological results was to directly compare the two UV reflectance metrics - one that fully included gloss and one that fully avoided it - and their persistent positive correlation (Fig. S6b-d) suggested that the overall measurement impact of glossiness was not the issue of greatest concern for the global dataset (although it did explain much residual variation specifically for the snakes with very low UV indices under manual scoring, low x-axis values in Fig S6b,c). However, further investigations of the impact of external scale morphology and especially oil content are critical for intersection with the studies of cellular mechanisms above.

Supplementary Note 2: Additional Quantitative Color Pattern Analysis (QCPA) parameters

Acuity correction

Type: Gaussian
Cycles per degree: 20
Viewing distance: 1m (1000, since units are in mm from scalebar)
Rescale to px per MRA: 5
Create luminance channel: See Supplementary Table S2 for photoreceptors used to generate luminance channels for each visual system.

RNL Ranked Filter

Weber fractions: See Supplementary Table S2 for visual-system-specific Weber fractions.
Luminance channel Weber fractions were always set to 0.1.
Iterations: 5
Radius: 5
Falloff: 3

RNL clustering

Colour JND Threshold: 2
Luminance JND Threshold: 3
Loops: 20
Radius multiplier: 2
Minimum cluster size: 10
Compare all clusters after pass: 6
Stop number: 1
Record from pass: 20

Weber fractions

Specific to visual system; see Supplementary Table 1 below.

Local Edge Intensity Analysis (LEIA)

Transformation: log
Ignore chromatic delta S values below: 0
Ignore achromatic delta S values below: 0

Supplementary Table 1. Visual system parameters used for visual system modeling. Plus symbols (+) indicate cone types that were used for luminance channels (if more than one is indicated, channels were averaged to create a luminance channel). LWS: long-wavelength-sensitive; MWS: medium-wavelength-sensitive; SWS: short-wavelength-sensitive; VS: violet-sensitive; UVS: ultraviolet-sensitive.

Visual system	Cone type	λ_{max}	Weber fraction	Lum. channel	UV trans. of ocular media	Citations
Dog	LWS	555	0.05	+	UV-transmissible lenses	Neitz & Geist (1989) Pretterer et al. (2004) Douglas & Jeffrey (2014)
	SWS	430	0.187	+		
Snake	LWS	555	0.05	+	UV-transmissible lenses	Simões et al. (2016)
	UVS	362	0.05			
Human	LWS	570	0.05	+	UV-blocking ocular media	Stockman & Sharpe (2000) Eisner & MacLeod (1980) Guth et al. (1968) Norren & Vos (1974)
	MWS	543	0.071	+		
	SWS	442	0.166			
Gecko	LWS	521	0.05	+	Transparent oil droplets are present, but transmit UV	Röll et al. (2000)
	MWS	464	0.05			
	UVS	362	0.05			
Peafowl	LWS	599	0.051		One oil droplet per cone type with 0.5 transmittance at 365nm; included in visual model	Hart (2002)
	MWS	537	0.05			
	SWS	477	0.054			
	VS	433	0.074			
	Double	567	0.100	+		
Bluetit	LWS	603	0.05		Similar to peafowl, but with 0.5 transmittance at 317nm; oil droplets included in visual model	Hart et al. (2000)
	MWS	541	0.05			
	SWS	453	0.059			
	UVS	372	0.082			
	Double	566	0.100	+		

Bibliography

1. Kikuchi DW, Seymoure BM, Pfennig DW. Mimicry's palette: widespread use of conserved pigments in the aposematic signals of snakes. *Evol & Develop* **16**, 61-67 (2014).
2. Akcali CK, Pfennig DW. Geographic variation in mimetic precision among different species of coral snake mimics. *J Evol Biol* **30**, 1420-1428 (2017).
3. Klein M-CG, Gorb SN. Ultrastructure and wear patterns of the ventral epidermis of four snake species (Squamata, Serpentes). *Zoology* **117**, 295-314 (2014).
4. Örnborg J, Andersson S, Griffith SC, Sheldon BC. Seasonal changes in a ultraviolet structural colour signal in blue tits,*Parus caeruleus*. *Biol J Linn Soc* **76**, 237-245 (2008).
5. Olsson M, Andersson S, Wapstra E. UV-deprived coloration reduces success in mate acquisition in male sand lizards (*Lacerta agilis*). *PLoS One* **6**, e19360 (2011).
6. Duellman WE. *Cusco Amazónico*. Comstock Pub. Associates (2005).
7. Saenko SV, Teyssier J, van der Marel D, Milinkovitch MC. Precise colocalization of interacting structural and pigmentary elements generates extensive color pattern variation in*Phelsuma*lizards. *BMC Biol* **11**, 1-13 (2013).
8. Mougeot F, Martinez-Padilla J, Pérez-Rodríguez L, Bortolotti GR. Carotenoid-based colouration and ultraviolet reflectance of the sexual ornaments of grouse. *Behav Ecol Sociobiol* **61**, 741-751 (2007).
9. Weller HI, Hiller AE, Lord NP, Van Belleghem SM. recolorize: An R package for flexible colour segmentation of biological images. *Ecol Lett* **27**, e14378 (2024).
10. Douglas RH, Jeffery G. The spectral transmission of ocular media suggests ultraviolet sensitivity is widespread among mammals. *Proc R Soc B* **281**, 20132995 (2014).
11. Eisner A, MacLeod DI. Blue-sensitive cones do not contribute to luminance. *JOSA* **70**, 121–123 (1980).
12. Guth S, Alexander J, Chumbly J, Gillman C, Patterson M. Factors affecting luminance additivity at threshold among normal and color-blind subjects and elaborations of a trichromatic-opponent colors theory. *Vision Res* **8**, 913–928 (1968).
13. Hart NS. Vision in the peafowl (Aves: Pavo cristatus). *J Exp Biol* **205**, 3925–3935 (2002).
14. Hart NS, Hunt DM. Avian visual pigments: Characteristics, spectral tuning, and evolution. *Am Nat* **169**, (2007).
15. Neitz J, Geist T, Jacobs GH. Color vision in the dog. *Visual Neurosci* **3**, 119-125 (1989).
16. Norren DV, Vos JJ. Spectral transmission of the human ocular media. *Vision Res* **14**, 1237-1244 (1974).
17. Pretterer G, Bubna-Littitz H, Windischbauer G, Gabler C, Griebel U. Brightness discrimination in the dog. *J Vision* **4**, 10–10 (2004).
18. Röll B. Characterization of retinal oil droplets in diurnal geckos (Reptilia, Gekkonidae). *J Exp Zool* **287**, 467–476 (2000).
19. Simões BF*, et al.* Visual pigments, ocular filters and the evolution of snake vision. *Mol Biol Evol* **33**, 2483–2495 (2016).
20. Stockman A, Sharpe LT. The spectral sensitivities of the middle-and long-wavelength-sensitive cones derived from measurements in observers of known genotype. *Vision Res* **40**, 1711–1737 (2000).
