# Supplementary material for: Ecological drivers of ultraviolet colour evolution in snakes
Source: Nat Commun. 2024 Jun 18;15:5213. doi: 10.1038/s41467-024-49506-4 (PMC11189474; doi:10.1038/s41467-024-49506-4)
Supplement: Supplementary file 3 — Description of Additional Supplementary Files [file 41467_2024_49506_MOESM3_ESM.pdf]

Description of Additional Supplementary Files

File Name: Supplementary Data 1
Description: Literature review categorisation scores, including title, keywords, and abstracts.

File Name: Supplementary Data 2
Description: Data file of all individuals sampled, museum collections and voucher number, sex, size, age, visible colours, and UV reflectance metrics for both human-scored and computer-scored approaches.

File Name: Supplementary Data 3
Description: Ecological habitat and diel activity scores, with references, for each species sampled.
