# Supplementary material for: Ecological drivers of ultraviolet colour evolution in snakes
Source: Nat Commun. 2024 Jun 18;15:5213. doi: 10.1038/s41467-024-49506-4 (PMC11189474; doi:10.1038/s41467-024-49506-4)
Supplement: Supplementary file 7 — Reporting Summary [file 41467_2024_49506_MOESM7_ESM.pdf]

nature portfolio

Corresponding author(s): Alison Davis Rabosky

Last updated by author(s): Mar 6, 2024

Reporting Summary

Nature Portfolio wishes to improve the reproducibility of the work that we publish. This form provides structure for consistency and transparency in reporting. For further information on Nature Portfolio policies, see our Editorial Policies and the Editorial Policy Checklist.

Statistics

For all statistical analyses, confirm that the following items are present in the figure legend, table legend, main text, or Methods section.

n/a	Confirmed	
☐	☒	The exact sample size (*n*) for each experimental group/condition, given as a discrete number and unit of measurement
☐	☒	A statement on whether measurements were taken from distinct samples or whether the same sample was measured repeatedly
☐	☒	The statistical test(s) used AND whether they are one- or two-sided<br>*Only common tests should be described solely by name; describe more complex techniques in the Methods section.*
☐	☒	A description of all covariates tested
☐	☒	A description of any assumptions or corrections, such as tests of normality and adjustment for multiple comparisons
☐	☒	A full description of the statistical parameters including central tendency (e.g. means) or other basic estimates (e.g. regression coefficient) AND variation (e.g. standard deviation) or associated estimates of uncertainty (e.g. confidence intervals)
☐	☒	For null hypothesis testing, the test statistic (e.g. *F*, *t*, *r*) with confidence intervals, effect sizes, degrees of freedom and *P* value noted<br>*Give P values as exact values whenever suitable.*
☒	☐	For Bayesian analysis, information on the choice of priors and Markov chain Monte Carlo settings
☒	☐	For hierarchical and complex designs, identification of the appropriate level for tests and full reporting of outcomes
☒	☐	Estimates of effect sizes (e.g. Cohen's *d*, Pearson's *r*), indicating how they were calculated

Our web collection on statistics for biologists contains articles on many of the points above.

Software and code

Policy information about availability of computer code

Data collection	Batch-Mask v.1.0.2
Data analysis	Software and packages used for this project are R v.4.2.1, micaToolbox v2.2.2, ImageJ v1.53T, 'phytools' v.1-2-0, and 'lmerTest' v.3.1-2. All code associated with data analyses are located in Deep Blue Data [https://doi.org/10.7302/2ktf-6k49].

For manuscripts utilizing custom algorithms or software that are central to the research but not yet described in published literature, software must be made available to editors and reviewers. We strongly encourage code deposition in a community repository (e.g. GitHub). See the Nature Portfolio guidelines for submitting code & software for further information.

Data

Policy information about availability of data

All manuscripts must include a data availability statement. This statement should provide the following information, where applicable:

- Accession codes, unique identifiers, or web links for publicly available datasets
- A description of any restrictions on data availability
- For clinical datasets or third party data, please ensure that the statement adheres to our policy

The database "Scopus" (https://www.scopus.com) was accessed for the UV function literature review. All raw data and materials are deposited in Deep Blue Data [https://doi.org/10.7302/2ktf-6k49] and all derivative data are in supplementary materials.

Research involving human participants, their data, or biological material

Policy information about studies with human participants or human data. See also policy information about sex, gender (identity/presentation), and sexual orientation and race, ethnicity and racism.

Reporting on sex and gender	N/A
Reporting on race, ethnicity, or other socially relevant groupings	N/A
Population characteristics	N/A
Recruitment	N/A
Ethics oversight	N/A

Note that full information on the approval of the study protocol must also be provided in the manuscript.

Field-specific reporting

Please select the one below that is the best fit for your research. If you are not sure, read the appropriate sections before making your selection.

☐ Life sciences ☐ Behavioural & social sciences ☒ Ecological, evolutionary & environmental sciences

For a reference copy of the document with all sections, see nature.com/documents/nr-reporting-summary-flat.pdf

Ecological, evolutionary & environmental sciences study design

All studies must disclose on these points even when the disclosure is negative.

Study description	We investigated the evolution of ultraviolet (UV) colouration across a broad range of snake species in the Western Hemisphere. First we assesed peer-reviewed literature on UV colouration in nature to quantify biases in reported function of UV colouration. To test the role of ecology on the evolution of UV colouration, we used both phylogenetic linear models and phylogenetic ANOVAs to analyze the effect of primary habitat usage (arboreal, terrestrial, fossorial, and aquatic) and activity patterns (nocturnal and diurnal) on amount of UV reflectance across snake body regions (N=104 species for one tree and 95 species for a second tree, which we used to assess the impact of phylogenetic uncertainty). To test for sex- and age-specific effects, we used mixed effect models with taxonomic Glade as a random effect (N=438 individuals from 5 clades). We ran each analysis as linear and multiple regression models to account for distribution non-normality. To assess interactions between body regions (e.g., dorsal vs. ventral, heads vs. bodies), we used Chi-square analyses for both individual data and species means. To test the relative effects of UV sensitivity and chromacy, we fit a multiple regression model predicting mean colour contrast.
Research sample	We captured 438 individual snakes (from 110 species) across the Western Hemisphere (Belize, Nicaragua, Peru, USA). We designed our sampling to capture the greatest species diversity and abundance possible, as snakes are highly secretive and difficult to sample. For most species, both male and female snakes were captured, as well as juvenile and adult snakes.
Sampling strategy	No sample size was predetermined; in order to maximize species diversity and sufficiently sample variation within species, we collected nearly all snakes that were encountered during each field excursion.
Data collection	We collected snakes through a combination of opportunistic foot surveys, pitfall traps, funnel traps, and driving surveys following the protocol of Davis Rabosky et al. 2021 (Biol. J. Linn. Soc.). We photographed each snake and took spectrometer measurements for a subset of individuals at each site at the time of capture before specimen preservation. All field work was conducted by H.L.C, J.D.C, and A.D.R. with associated field teams (see Acknowledgments).
Timing and spatial scale	We collected snakes throughout multiple, multi-week field expeditions between the years 2016-2021. Field sampling locations were Estaciones Biologicos de Los Amigos (Madre de Dios), Villa Carmen (Cusco), Madre Selva (Loreto), and Santa Cruz (Loreto) in Peru (2016-2018); Las Brisas del Mogoton, Laguna de Asososca, and Rio San Juan in Nicaragua (May-June 2018); Foundation for Research and Environmental Education (BFREE) Field Station and Douglas Da Silva Forest Station in Belize (May-June 2019); southern Texas, USA (May 2021); and Colorado, USA (May-June 2021).
Data exclusions	We excluded snakes from all colour analyses if their photographs were too poor-quality to accurately score (i.e., under/over exposed, blurry), and we excluded species from phylogenetic analysis if they were not represented by molecular data in a phylogeny.
Reproducibility	Our study did not involve an experimental manipulation of organisms. Human observations of snake colors and categorization of UV-function in the literature review were conducted in a double-blind set up to ensure scoring was consistent and reproducible. All code for analyses was run by at least two authors to ensure repeatability.
Randomization	Randomization is not relevant to our study because we did not experimentally manipulate organisms.

Blinding	We used human-scored snake ultraviolet colouration through a double-blind system to reduce biases in colour assessment and data collection. We did not use blinding in any other portion of this study.

Did the study involve field work? ☒ Yes ☐ No

Field work, collection and transport

Field conditions	Field conditions varied greatly between sites. We sampled in the lowland and foothill rainforests of Peru, Belize, and Nicaragua across the wet and dry seasons, as well as in open desert and arid mountain ranges in Texas and Colorado, USA, respectively.
Location	We sampled at four sites in Peru: Los Amigos (-12.569041, -70.100926), Villa Carmen (-12.895169, -71.406245), Madre Selva (-3.62225, -72.2465), and Santa Cruz (-3.5285, -73.1790). We sampled at three sites in Nicaragua: Las Brisas del Mogoton (13.7402, -86.3790), Laguna de Asososca (12.4272, -86.6613), and Rio San Juan (10.9776, -84.3337). We sampled at two sites in Belize: Foundation for Research and Environmental Education (BFREE) Field Station (16.5519, -88.7077) and Douglas Da Silva Forest Station (16.9790, -89.0066). We sampled at three sites in Texas, USA: Richland Creek Wildlife Management Area (31.9288, -96.0587), Hidalgo County (26.3146, -98.2730), and Big Bend National Park (30.1671, -103.2376). We sampled at one site in Colorado: Rattlesnake Butte (40.573287, -107.017650).
Access & import/export	Access to field sites and export of specimens were approved by respective governmental authorities (Peru: Servicio Nacional Forestal y de Fauna Silvestre 029-2016-SERFOR-DGGSPFFS, 405-2016-SERFOR-DGGSPFFS, 116-2017-SERFOR-DGGSPFFS; Nicaragua: Ministerio del Ambiente y los Recursos Naturales DGB-IC-058-2017, DGPNB-IC-019-2018, DGPNB- IC-020-2018, DGPNB-IC-002-2019; Belize: Forest Department of the Ministry of Agriculture, Fisheries, Forestry, the Environment and Sustainable Development FD/WL/1/19(10); Texas Parks and Wildlife, #SPR-1020-175; Colorado Department of Natural Resources, #1950298916).
Disturbance	This project was conducted with minimal to no disturbance to the surrounding sampling environment. We collected snakes through a combination of opportunistic foot surveys, pitfall traps, funnel traps, and driving surveys following the protocol of Davis Rabosky et al. 2021 (Biol. J. Linn. Soc.). We removed all traps at the end of every sampling event at each site.

Reporting for specific materials, systems and methods

We require information from authors about some types of materials, experimental systems and methods used in many studies. Here, indicate whether each material, system or method listed is relevant to your study. If you are not sure if a list item applies to your research, read the appropriate section before selecting a response.

Materials & experimental systems

n/a	Involved in the study	
☒	☐	Antibodies
☒	☐	Eukaryotic cell lines
☒	☐	Palaeontology and archaeology
☒	☐	Animals and other organisms
☒	☐	Clinical data
☒	☐	Dual use research of concern
☒	☐	Plants

Methods

n/a	Involved in the study	
☒	☐	ChIP-seq
☒	☐	Flow cytometry
☒	☐	MRI-based neuroimaging

Plants

Seed stocks	N/A
Novel plant genotypes	N/A
Authentication	N/A
